# HDL Interfere with the Binding of T Cell Microparticles to Human Monocytes to Inhibit Pro-Inflammatory Cytokine Production

**DOI:** 10.1371/journal.pone.0011869

**Published:** 2010-07-29

**Authors:** Rakel Carpintero, Lyssia Gruaz, Karim J. Brandt, Anna Scanu, Dorothée Faille, Valery Combes, Georges E. Grau, Danielle Burger

**Affiliations:** 1 Hans Wilsdorf Laboratory, Inflammation and Allergy Research Group, Division of Immunology and Allergy, Department of Internal Medicine, Faculty of Medicine and University Hospital, University of Geneva, Geneva, Switzerland; 2 Department of Clinical and Experimental Medicine, University of Padova, Padova, Italy; 3 Department of Pathology, University of Sydney, Camperdown, Australia; New York University, United States of America

## Abstract

**Background:**

Direct cellular contact with stimulated T cells is a potent mechanism that induces cytokine production in human monocytes in the absence of an infectious agent. This mechanism is likely to be relevant to T cell-mediated inflammatory diseases such as rheumatoid arthritis and multiple sclerosis. Microparticles (MP) generated by stimulated T cells (MP_T_) display similar monocyte activating ability to whole T cells, isolated T cell membranes, or solubilized T cell membranes. We previously demonstrated that high-density lipoproteins (HDL) inhibited T cell contact- and MP_T_-induced production of IL-1β but not of its natural inhibitor, the secreted form of IL-1 receptor antagonist (sIL-1Ra).

**Methodology/Principal Findings:**

Labeled MP_T_ were used to assess their interaction with monocytes and T lymphocytes by flow cytometry. Similarly, interactions of labeled HDL with monocytes and MP_T_ were assessed by flow cytometry. In parallel, the MP_T_-induction of IL-1β and sIL-1Ra production in human monocytes and the effect of HDL were assessed in cell cultures. The results show that MP_T_, but not MP generated by activated endothelial cells, bond monocytes to trigger cytokine production. MP_T_ did not bind T cells. The inhibition of IL-1β production by HDL correlated with the inhibition of MP_T_ binding to monocytes. HDL interacted with MP_T_ rather than with monocytes suggesting that they bound the activating factor(s) of T cell surface. Furthermore, prototypical pro-inflammatory cytokines and chemokines such as TNF, IL-6, IL-8, CCL3 and CCL4 displayed a pattern of production induced by MP_T_ and inhibition by HDL similar to IL-1β, whereas the production of CCL2, like that of sIL-1Ra, was not inhibited by HDL.

**Conclusions/Significance:**

HDL inhibit both MP_T_ binding to monocytes and the MP_T_-induced production of some but not all cytokines, shedding new light on the mechanism by which HDL display their anti-inflammatory functions.

## Introduction

An unbalanced cytokine homeostasis plays an important part in the pathogenesis of chronic inflammatory diseases. This suggests that the mechanisms ruling the production of pro-inflammatory cytokines, their inhibitors, and inhibitory mechanisms escape normal controls. IL-1β is a prototypical pro-inflammatory cytokine whose involvement in immuno-inflammatory diseases such as multiple sclerosis (MS) and rheumatoid arthritis (RA) is well established. In the absence of an infectious agent (*i.e.*, in non-septic conditions), the nature of the factors triggering the production of the prototypical pro-inflammatory cytokines, TNF and IL-1β, is still elusive. In chronic inflammatory diseases of autoimmune etiology, T cells and monocytes/macrophages infiltrate the target tissue. In animal models of MS and RA, the transfer of T cells isolated from diseased animals induces the disease in healthy animals, strongly suggesting that T cells play a pathogenic role [1], [2]. It is now acknowledged that direct cellular contact with stimulated T cells induces the massive up-regulation of IL-1 and TNF in human monocytes/macrophages [3]–[5]. Besides triggering pro-inflammatory cytokine production, contact-mediated activation of monocytes also induces the production and/or shedding of cytokine inhibitors such as the secreted form of IL-1 receptor antagonist (sIL-1Ra), and soluble receptors of IL-1 and TNF [6]–[9]. Once stimulated, most T cell types, including T cell clones, freshly isolated T lymphocytes, and T cell lines such as HUT-78 cells, induce the production of IL-1β and TNF in monocytes/macrophages [10]. Furthermore, depending on T cell type and T cell stimulus, direct cellular contact with stimulated T lymphocytes can induce different patterns of products in monocytes/macrophages (reviewed in [3], [4], [11]), suggesting that multiple ligands and counter-ligands are involved in the contact-mediated activation of monocytes/macrophages. This premise strengthened by observations showing that Th1 cell clones preferentially induce IL-1β rather than sIL-1Ra production, and cytokine-stimulated T lymphocytes induce TNF production while failing to trigger that of IL-10 [8], [12]. Therefore, cellular contact with stimulated T cells can induce an imbalance in the production of pro-inflammatory versus anti-inflammatory cytokines, reflecting that observed in chronic inflammatory diseases.

By generating microparticles (MP) cells can disseminate cell surface molecules and thus ensure “distant” cellular contact. MP are fragments (0.1–1 µm diameter) shed from the plasma membrane of stimulated or apoptotic cells. Having long been considered inert debris reflecting cellular activation or damage, MP are now acknowledged as cellular effectors involved in cell-cell crosstalk [13]. Indeed, MP display membrane proteins as well as bioactive lipids implicated in a variety of fundamental processes and thus constitute a disseminated pool of bioactive effectors [14]. MP are present in the circulation of healthy subjects, and their numbers increase upon various pathological conditions [15]. Elevated MP have also been reported in chronic inflammatory diseases [16]–[18] including RA [19]–[22] and MS [18], [23]–[26]. Although present in patients' plasma, MS cerebrospinal fluid has, to our knowledge, not been investigated for the presence of MP. In RA synovial fluid, MP are abundant and modulate fibroblast-like synoviocyte activity *in vitro* [21], [22], [27], [28]. We recently demonstrated that MP generated by stimulated T cells can activate monocytes to produce cytokines similarly to membranes or solubilized membranes of stimulated T cells [29]. Furthermore, T cell contact-induced production of IL-1β and TNF in monocytes is specifically inhibited by high-density lipoproteins (HDL)-associated apolipoprotein A–I (apo A–I) [30], a “negative” acute-phase protein. HDL may infiltrate the inflamed tissue to counteract T cell contact-induce monocytes activation [31]. Furthermore, microarray analysis demonstrated that direct contact with stimulated T cells induces the expression of genes mostly related to inflammatory pathways but different from those induced under acute/infectious inflammatory conditions (e.g., induced by lipopolysaccharides), and that HDL inhibit the expression of pro rather than anti-inflammatory molecules [32]. For instance, in contrast to the production of IL-1β, HDL do not inhibit that of sIL-1Ra [29]. However, the mechanism by which HDL affect cytokine production in monocytes is still elusive. In this study we used MP to assess their interaction with monocytes and the effects of HDL. The results show that MP generated by stimulated T cells bind monocytes but not T lymphocytes and that HDL inhibit the interaction of MP_T_ with monocytes. Therefore, HDL may inhibit cytokine production in human monocytes by interfering with the binding of the activating factor(s) at the surface of stimulated T cells to receptor(s) at the surface of monocytes.

## Results

### Characterization of microparticles generated by stimulated HUT-78 cells (MP_T_)

**Figure 1:**
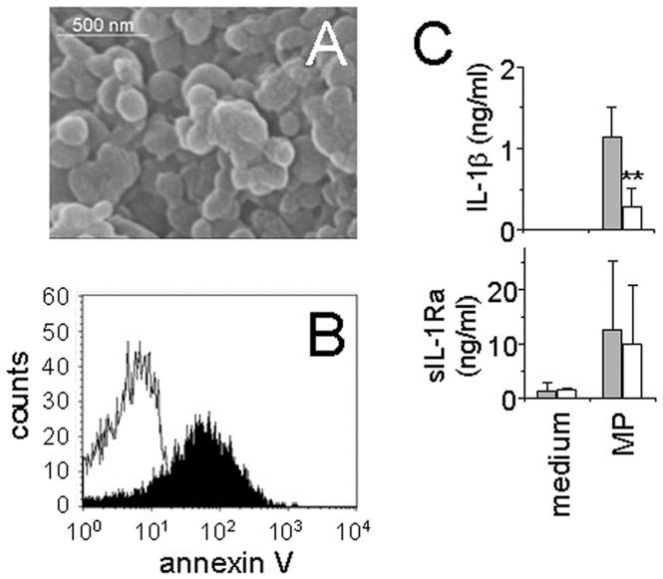
Characterization of microparticles generated by stimulated HUT-78 cells (MP_T_). (A) Scanning electron microscopy of isolated MP_T_. Scale bar  = 500 nm. (B) Flow cytometry analysis of the binding of FITC-annexin V to MP_T_. (C) Monocytes (5×10^4^ cells/200 µl/well; 96-well plates) were activated by MP_T_ (3 µg/ml) for 24 h in the presence (empty columns) or absence (grey columns) of HDL (0.2 mg/ml proteins). Cell culture supernatants were measured for the presence of the indicated cytokines. Results are expressed as mean ± SD of 3 experiments carried out with monocytes isolated from 3 individual donors. ** p<0.01, as determined by paired student t test.

We previously demonstrated that MP generated by stimulated HUT-78 cells (here referred to as MP_T_) display similar monocyte activating ability to MP generated by stimulated blood T lymphocytes [29]. In the present study we used MP_T_ to avoid variations often observed between T lymphocytes from different blood donors. Prior to assessing the ability of MP_T_ to activate human monocytes, we determined their physicochemical characteristics. As demonstrated by electron microscopy, MP_T_ are round particles with heterogeneous sizes displaying diameters between 0.1 and 0.8 µm, although most of MP_T_ were of small size (Fig. 1A). Flow cytometry analysis of MP_T_ preparation shows that particles between 0.1 and 0.8 µm bound annexin V (Fig. 1B) demonstrating that phosphatidylserine was exposed at their surface, thus defining them as microparticles. To assess the quality of MP_T_ preparations, we tested their ability to activate IL-1β and sIL-1Ra production in isolated monocytes. As previously described [29], MP isolated from unstimulated HUT-78 cells did not affect the production of cytokines in human monocytes (data not shown). We previously determined that the production of both IL-1β and sIL-1Ra was induced in a dose-response manner by MP_T_, the production of sIL-1Ra reaching a plateau at 1 µg/ml proteins of MP_T_ while that of IL-1β was still increasing at 6 µg/ml proteins of MP_T_ [29]. Here we used an intermediate dose, 3 µg/ml proteins of MP_T_, which induced the production of both IL-1β and sIL-1Ra in monocytes (Fig. 1C). MP_T_-induced IL-1β production was inhibited in the presence of 0.2 mg/ml HDL, *i.e.*, a concentration that was determined to be optimal [30]. In contrast, sIL-1Ra production was not significantly affected by HDL, suggesting that different pathways or surface molecules were involved in the induction of the latter molecules. These results demonstrate that MP_T_ were able to activate monocytes and confirmed previous results suggesting that HDL inhibited only a part of factors induced by contact with stimulated T cells or MP_T_ [29], [32].

### MP_T_ specifically bind and activate human monocytes

**Figure 2:**
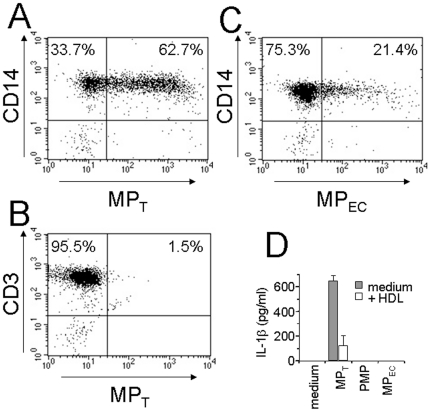
MP_T_ specifically bind and activate human monocytes. (A–C) The binding of PKH67-labelled MP from different cellular sources to isolated human monocytes and T lymphocytes was assessed by flow cytometry. Binding of MP_T_ (12 µg/ml) to CD14^+^ monocytes (A) and CD3^+^ T lymphocytes (B). (C) Binding of endothelial cell MP (MP_EC_; 12 µg/ml) to CD14^+^ monocytes. (D) Monocytes (5×10^4^ cells/well/200 µl/well; 96-well plates) were activated by 3 µg/ml MP_T_, 14 µg/ml activated endothelial cells (MP_EC_) and 14 µg/ml activated platelets (PMP) in the presence (empty columns) or absence (grey columns) of 0.2 mg/ml HDL. IL-1β was measured in culture supernatants after 24 h incubation. Results are expressed as mean ± SD of triplicates.

Since direct cellular contact with stimulated T cells is required to induce cytokine production in monocytes [33], we sought to assess whether MP_T_ were able to durably interact with monocytes. To this aim, we assessed the binding of green PKH67-labelled MP_T_ to CD14^+^ monocytes by flow cytometry. A large part of CD14^+^ monocytes (62.7%) bound MP_T_ (Fig. 2A). Non-specific MP_T_ binding to or fusion with target cell membranes was ruled out since MP_T_ did not bind CD3^+^ cells, *i.e.*, lymphocytes (Fig. 2B). This suggests that MP_T_ specifically interacted with monocytes. Furthermore, MP isolated from supernatants of unstimulated HUT-78 cells did not bind to CD14^+^ monocytes (data not shown), further suggesting that the binding of MP_T_ to monocytes occurred through molecules expressed at the surface of stimulated T cells but not on unstimulated cells. A fraction of CD14^+^ monocytes (21.4%) bound MP from TNF-activated endothelial cells but were not induced to produce IL-1β (Figs. 2C and 2D). Indeed, only MP_T_ triggered the production of IL-1β in human monocytes, whereas MP generated from activated platelets or endothelial cells were inefficient, even at concentrations 4- to 5-fold higher than that of MP_T_ (Fig. 2D). Together these results suggest that only MP_T_ were able to bind and activate monocytes to produce IL-1β.

### HDL inhibit MP_T_ interactions with human monocytes

**Figure 3:**
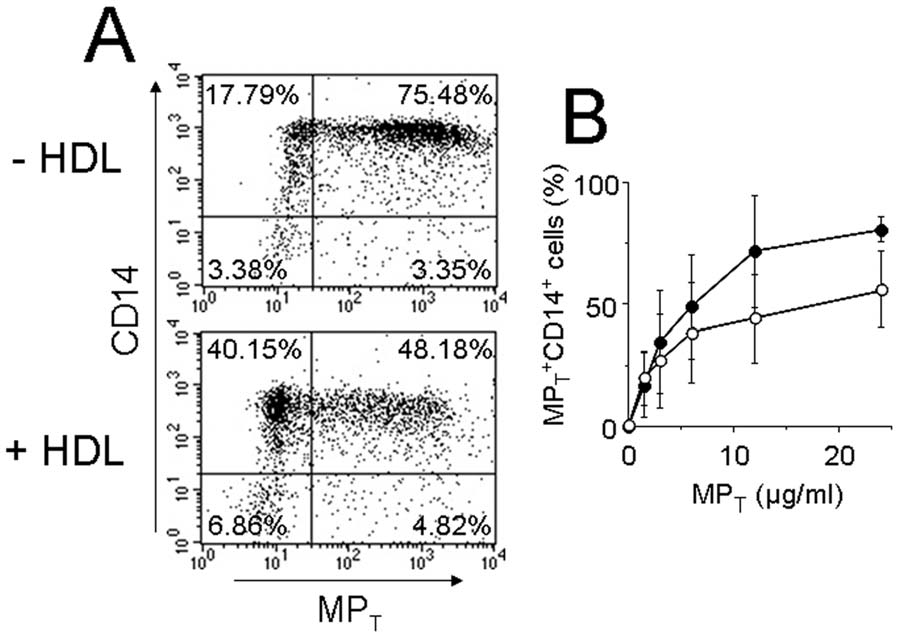
HDL inhibit the binding of MP_T_ to human monocytes. The binding of PKH67-labelled MP_T_ to CD14^+^ monocytes in the presence or absence of HDL was measured by flow cytometry. (A) Representative binding of PKH67-labelled MP_T_ (12 µg/ml proteins) to CD14^+^ monocytes in the presence or absence of 0.2 mg/ml HDL (as indicated). (B) Flow cytometry measurement of the binding of increasing concentration of PKH67-labelled MP_T_ to CD14^+^ monocytes in the absence (closed circles) or presence (empty circles) of 0.2 mg/ml HDL. The percentage ± SD of MP_T_^+^CD14^+^ monocytes (upper right panel) in 3 different experiments is presented.

Because HDL inhibited IL-1β production in MP_T_-activated monocytes, we assessed whether they would interfere with MP_T_ binding to monocytes. As shown in Fig. 3A, the binding of MP_T_ (12 µg/ml) to monocytes was inhibited in the presence of 0.2 mg/ml HDL. The binding of PKH67-labelled MP_T_ was dose-dependent and reached a plateau between 12 and 24 µg/ml protein, *i.e.*, around 1×10^6^ MP/ml (Fig. 3B). HDL inhibited the binding of MP_T_ to monocytes by 30±12% between 3 and 24 µg/ml MP_T_ (Fig. 3B). This observation suggests that HDL inhibit IL-1β production by interfering with the binding of the activating factor to its receptor on monocytes.

### HDL bind MP_T_

**Figure 4:**
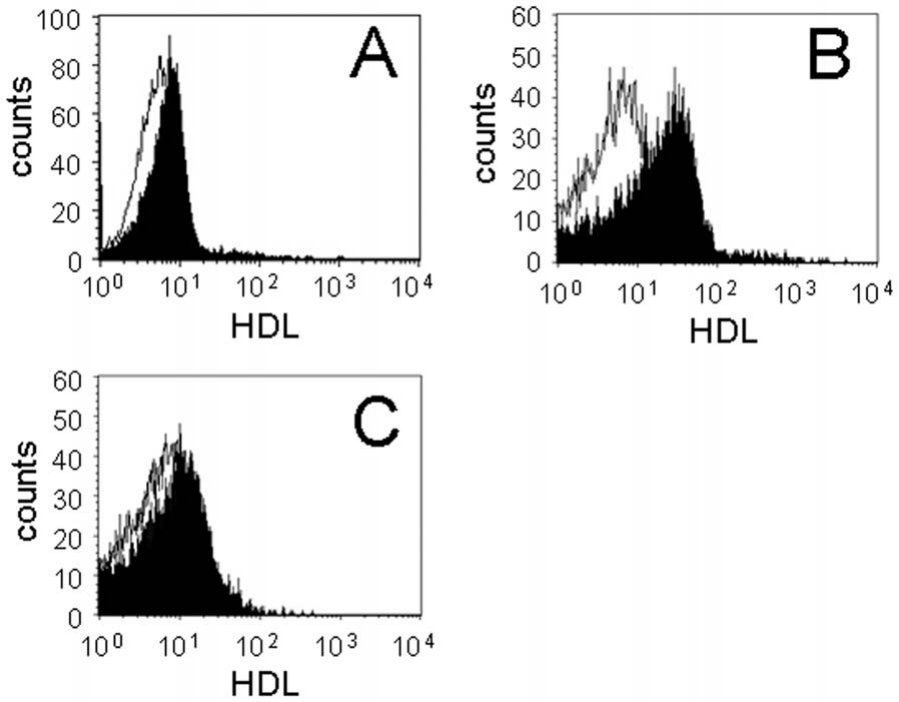
HDL interaction with MP_T_. The binding of FITC-HDL to monocytes (A), MP_T_ (B) and MP from unstimulated HUT-78 cells (C) was analyzed by flow cytometry. Results are representative of 3 different experiments.

To determine whether HDL interacted with the activating factor on MP_T_ or to its monocytic receptor, the binding of FITC-HDL to monocytes and MP from both stimulated and resting HUT-78 cells was assessed by flow cytometry. FITC-HDL bound CD14^+^ monocytes to some extent, a small enhancement of fluorescence intensity being observed (Fig. 4A), confirming previous results [30]. In contrast, FITC-HDL bound MP_T_ to a great extent (Fig. 4B) suggesting that HDL might inhibit monocyte activation by primarily interacting with the activating factor(s) at the surface of MP_T_, *i.e.*, at the surface of stimulated T cells. Interestingly, FITC-HDL only slightly interacted with MP isolated from unstimulated T cells (Fig. 4C), indicating that HDL bound to molecules that were only expressed on stimulated T cells. Together these results show that HDL are likely to inhibit the production of cytokines in monocytes activated by MP_T_ by competing with the monocyte receptor(s) for binding the activating factor.

### HDL inhibit MP_T_-induced cytokine and chemokine production in human monocytes

**Figure 5:**
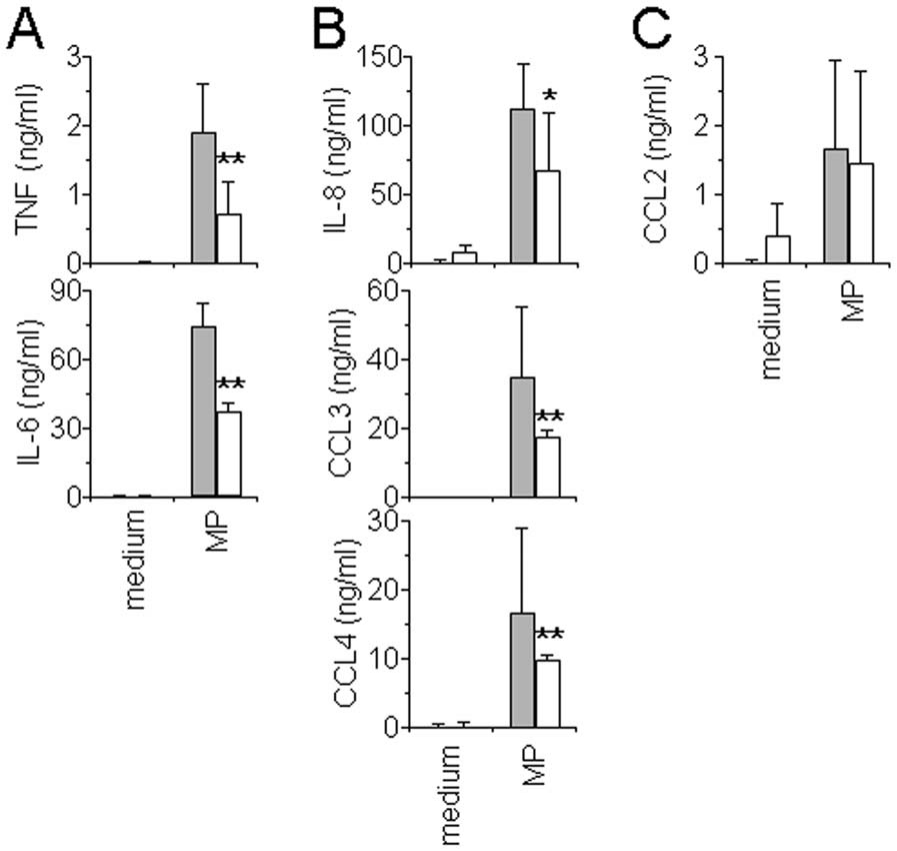
Modulation of cytokine production by HDL in MP_T_-activated monocytes. Monocytes (5×10^4^ cells/200 µl/well; 96-well plates) were activated by MP_T_ (6 µg/ml) for 24 h in the presence (empty columns) or absence (grey columns) of HDL (0.2 mg/ml proteins). Cell culture supernatants were measured for the presence of the indicated cytokines. Results are expressed as mean ± SD of 3 experiments carried out with monocytes isolated from 3 individual donors. * p<0.05; ** p<0.01, as determined by paired student t test.

HDL are not a general inhibitor of T cell contact-activation of human monocytes [32]. Indeed, HDL preferentially inhibited the expression of factors with a pro-inflammatory profile, as exemplified by IL-1β, in the present study, whilst they did not affect the expression of anti-inflammatory factors, exemplified here by sIL-1Ra. To extend this observation to the effect of HDL on MP_T_-induced cytokine production in human monocytes, we assessed the effects of HDL on a range of cytokines and chemokines induced by MP_T_ in human monocytes. As shown in Fig. 5, in addition to that of IL-1β and sIL-1Ra, MP_T_ induced the production of the prototypical pro-inflammatory cytokines TNF and IL-6, and the chemokines IL-8, CCL2, CCL3 and CCL4. The production of pro-inflammatory cytokines was inhibited in the presence of HDL (Fig. 5A) suggesting that they were induced by a similar activating factor as the one inducing IL-1β production. This was also true for chemokines (Fig. 5B), with the exception of CCL2 (Fig. 5C), whose production was not affected by HDL similarly to that of sIL-1Ra. By comparison with results obtained in monocytes activated by CE_sHUT_ [32], the present data demonstrate that MP_T_ indeed displayed similar activity as soluble extracts of membranes isolated from stimulated HUT-78 cells, *i.e.*, CE_sHUT_. Furthermore they strengthen results of Fig. 4 demonstrating that different surface molecules were involved in monocyte activation, part of them being inhibited through interaction with HDL.

## Discussion

This study reveals that MP_T_ specifically interact with monocytes to trigger cytokine and chemokine production. MP_T_-monocyte interaction is inhibited by HDL which are likely to bind the activating factor(s) on MP_T_, in turn inhibiting pro-inflammatory cytokine and chemokine production in monocytes. Interestingly, the production of sIL-1Ra and CCL2 was not inhibited in the presence of HDL confirming previous results [29], [32] and suggesting that different factors at the surface of stimulated T cells and MP_T_ are involved in the induction of pro- and anti-inflammatory factors in monocytes.

Although studies showed that MP from endothelial cells and platelets could induce the expression of adhesion molecules and tissue factor-dependent procoagulant activity in the monocytic cell line THP-1 [34], [35], activation of freshly isolated monocytes is not a general characteristic of MP in terms of induction of cytokine production. Indeed, MP generated by activated endothelial cells and platelets do not induce IL-1β production in monocytes. However, a small percentage of monocytes do bind MP from endothelial cells, demonstrating that MP interaction with monocytes is not exclusively due to interactions between activating factors at the surface of MP_T_ and receptors/counter-ligands on monocytes, but may occur through adhesion molecules likely to be present on the surface of all MP as demonstrated in MP generated by endothelial cells and neutrophils [36], [37]. This suggests that the binding of MP to target cells may occur through multiple ligands and counter-ligands. It is likely to be the case for MP_T_, since only part of their binding to monocytes is inhibited in the presence of HDL indicating that interactions occur through ligands different from the IL-1β activating factor(s). Partial inhibition of MP_T_ binding to monocytes by HDL is also reflected by the inhibition of the production of a part of cytokines and chemokines induced by MP_T_ (see Fig. 5), suggesting the involvement of activating factors which do not bind and therefore are not inhibited by HDL as exemplified by sIL-1Ra and CCL2 in the present study.

HDL do not represent a universal inhibitor of monocyte activation since they inhibit the production of only particular factors induced by contact with MP_T_. Indeed, among the cytokines and chemokines which production is induced in monocytes upon contact with MP_T_, sIL-1Ra and CCL2 are not inhibited by HDL. These results are reminiscent of previous data showing that the production of sIL-1Ra, CCL2, and other factors that mainly display anti-inflammatory functions, is not inhibited by HDL upon activation by CE_sHUT_ [32]. Indeed, HDL mainly inhibit pro-inflammatory pathways induced by contact with stimulated T cells. CCL2 which is a major monocyte chemoattractant is far to be a prototypical pro-inflammatory factor. Indeed, CCL2 influences T cell immunity in that it induces a bias towards Th2 polarization [38]. Because chronic inflammatory diseases such as MS and RA in which T cell contact is likely to play a pathogenic part are mediated by Th1 and Th17, the production of CCL2 by monocytes/macrophages might be considered as an attempt to revert T cell polarization to a less inflammatory phenotype [39]. Besides, the premise that the activation of cytokine production by CE_sHUT_ and MP_T_ is similarly inhibited by HDL, confirms that MP_T_ and stimulated T cells exhibit similar surface molecules. In agreement with this observation, multiple studies have shown that MP express similar surface proteins to the cell they originate from (reviewed in [40]). Since HDL bind activating factor(s) at the surface of stimulated T cells and MP_T_, it is likely that different molecules on T cells activate monocytes to secrete cytokines and chemokines; the activity of some/one of them being inhibited by HDL.

In conclusion, this study demonstrates that stimulated T cells and MP_T_ express surface factor(s) that bind monocytes and in turn induce cytokine production. Both MP_T_ binding and the MP_T_-induced production of some but not all cytokines are inhibited by HDL, suggesting that different factors at the surface of T cells and MP_T_ trigger the production of cytokines. Although the identity of the activating factors remains elusive, the premise that it displays tight interactions with monocytes and HDL may provides clues as to its identification.

## Materials and Methods

### Ethics statement

Buffy coats of blood of healthy donors were provided by the Geneva Hospital Blood Transfusion Center. In accordance with the ethical committee of the Geneva Hospital, the blood bank obtained informed consent from the donors, who are thus informed that part of their blood will be used for research purposes.

### Materials

Fetal calf serum (FCS), streptomycin, penicillin, L-glutamine, RPMI-1640 and PBS free of Ca^2+^ and Mg^2+^ (Gibco, Paisley, Scotland); purified phytohaemagglutinin (PHA) (EY Laboratories, San Marco, CA); Ficoll-Paque (Pharmacia Biotech, Uppsala, Sweden); phorbol myristate acetate (PMA), phenylmethylsulfonyl fluoride (PMSF), polymyxin B sulfate, amphiphilic cell linker dye kit (PKH67), calcium ionophore A23187, human TNF, and bovine serum albumin (Sigma Chemicals Co., St. Louis, MO); and annexin V-FITC, PE-labeled anti-human CD14, and anti-human CD3 (BD Biosciences) were purchased from the designated suppliers. Other reagents were of analytical grade or better.

### Blood monocytes and T lymphocytes

Peripheral blood monocytes and T lymphocytes were isolated from buffy coats of blood of healthy volunteers as previously described [30]. In order to avoid activation by endotoxin, polymyxin B (2 µg/ml) was added to all solutions during the monocyte isolation procedure.

### T cell stimulation and Isolation and labeling of microparticles (MP)

The human T cell line HUT-78 was purchased from the ATCC (Rockville, MD). Cells were maintained in RPMI-1640 medium supplemented with 10% heat-inactivated FCS, 50 µg/ml streptomycin, 50 U/ml penicillin and 2 mM L-glutamine in 5% CO_2_-air humidified atmosphere at 37°C. HUT-78 cells (2×10^6^ cells/ml) were stimulated for 6 h with PHA (1 µg/ml) and PMA (5 ng/ml) as previously described [41], [42]. MP were isolated from culture supernatants of HUT-78 cells as previously described [29]. MP isolated from supernatants of stimulated HUT-78 cells were referred to as MP_T_. As previously demonstrated, MP_T_ display similar cytokine induction ability as MP generated by stimulated T lymphocytes isolated from human blood [29]. Total RNA in MP_T_ reached 35.2±17.5 µg/mg proteins, i.e., 0.7±0.4 µg RNA/10^6^ MP_T_. This suggests that MP_T_ were indeed closed vesicles able to protect RNA from degradation by RNases. IL-1β and sIL-1Ra were not detected in MP_T_ or MP from unstimulated HUT-78 cells. DNA was below the detection limit, thus amounting to <3 ng/mg proteins in MP_T_, suggesting that no or few apoptotic bodies were present amongst MP_T_. Alternatively, MP were isolated from culture supernatants of human brain endothelial cells activated with TNF (MP_EC_) and human blood platelets activated with the ionophore A23187 (PMP) as described previously [16], [43]. Isolated MP were counted and their protein content measured as described [29]. MP preparations contained 19.7±4.2 µg proteins/10^6^ MP independently of the cellular origin confirming previous results [29]. MP were labeled with a green fluorescent amphiphilic cell linker dye kit (PKH67, Sigma) as described elsewhere [43].

### Scanning electron microscopy (SEM)

MP_T_ were centrifuged (20,000 g, for 45 min) and the pellet fixed with 2% glutaraldehyde (Sigma) in 0.1 M sodium cacodylate, pH 7.4. The fixed MP_T_ were treated with 1% osmium tetroxide (Sigma) in 0.1 M cacodylate buffer prior to dehydration in increasing concentrations of ethanol (30 to 100%). MP_T_ were then critical-point dried, sputter-coated with gold, and observed under a Cambridge Stereoscan 260 scanning electron microscope.

### Isolation, labeling and immobilization of HDL

Human serum HDL were isolated according to Havel et al. [44]. When required, HDL were labeled with fluorescein isothiocyanate (FITC-HDL) as previously described [30]. The binding of FITC-HDL to cells and MP_T_ was analyzed by direct flow cytometry on a flow cytometer (FACSCalibur, BD) as previously described [30].

### Cytokine production and measurement

Monocytes (5×10^4^ cells/well/200 µl) were activated with the indicated stimulus in RPMI 1640 medium supplemented with 10% heat-inactivated FCS, 50 µg/ml streptomycin, 50 U/ml penicillin, 2 mM L-glutamine and 5 µg/ml polymyxin B sulfate (medium) in 96 well plates and cultured for 24 h unless stated otherwise. When required, monocytes (2×10^6^ cells/well/1 ml) were pre-activated by MP_T_ (6 µg/ml) in 24-well Ultra Low Attachment plates (Corning). After the indicated time, cells were harvested, washed in PBS and then activated as described above. The production of cytokines was measured in culture supernatants by commercially available enzyme immunoassay: IL-1β (Beckman Coulter Inc.), other cytokines and chemokines (Quantikine, R&D, Minneapolis, MN).

### MP binding to target cells

Monocytes or T lymphocytes (2×10^5^ cells/well/200 µl) were incubated for 3 h at 37°C with the indicated concentration of PKH67-labelled MP in round bottom polypropylene 96-well plates. After washing with PBS containing 2% heat inactivated human AB serum, 1% BSA and 0.1% NaN_3_, cells were incubated with PE-labeled anti-human CD14 (monocytes) or anti-human CD3 (T lymphocytes) antibodies for 20 min. After thorough washing, cells were analyzed by flow cytometry (FACSCalibur, BD). Buffers used for flow cytometry analysis were subjected to filtration (Stericup 0.22 µm, Millipore) to discard interferences with small debris.

### Statistics

When required, significance of differences between groups was evaluated using Student's paired *t* test.

## References

[1] Paterson PY (1960). Transfer of allergic encephalomyelitis in rats by means of lymph node cells.. J Exp Med.

[2] Pearson CM, Wood FD (1964). Passive transfer of adjuvant arthritis by lymph node or spleen cells.. J Exp Med.

[3] Brennan FM, Foey AD (2002). Cytokine regulation in RA synovial tissue: role of T cell/macrophage contact-dependent interactions.. Arthritis Res.

[4] Burger D, Dayer JM, Molnarfi N, Smolen JS, Lipsky PE (2007). Cell contact dependence of inflammatory events.. Contemporary Targeted Therapies in Rheumatology.

[5] Li YY, Bao M, Meurer J, Skuballa W, Bauman JG (2008). The identification of a small molecule inhibitor that specifically reduces T cell-mediated adaptive but not LPS-mediated innate immunity by T cell membrane-monocyte contact bioassay.. Immunol Lett.

[6] Vey E, Dayer JM, Burger D (1997). Direct contact with stimulated T cells induces the expression of IL-1β and IL-1 receptor antagonist in human monocytes. Involvement of serine/threonine phosphatases in differential regulation.. Cytokine.

[7] Vey E, Burger D, Dayer JM (1996). Expression and cleavage of tumor necrosis factor-α and tumor necrosis factor receptors by human monocytic cell lines upon direct contact with stimulated T cells.. Eur J Immunol.

[8] Chizzolini C, Chicheportiche R, Burger D, Dayer JM (1997). Human Th1 cells preferentially induce interleukin (IL)-1β while Th2 cells induce IL-1 receptor antagonist production upon cell/cell contact with monocytes.. Eur J Immunol.

[9] Coclet-Ninin J, Dayer JM, Burger D (1997). Interferon-β not only inhibits interleukin-1 β and tumor necrosis factor-α but stimulates interleukin-1 receptor antagonist production in human peripheral blood mononuclear cells.. Eur Cytokine Netw.

[10] Burger D, Roux-Lombard P, Chizzolini C, Dayer JM, van den Berg WB, Miossec P (2004). Cell-cell contact in chronic inflammation: the importance to cytokine regulation in tissue destruction and repair.. Cytokines and Joint Injury.

[11] Burger D (2000). Cell contact-mediated signaling of monocytes by stimulated T cells: a major pathway for cytokine induction.. Eur Cytokine Netw.

[12] Sebbag M, Parry SL, Brennan FM, Feldmann M (1997). Cytokine stimulation of T lymphocytes regulates their capacity to induce monocyte production of tumor necrosis factor-α, but not interleukin-10: Possible relevance to pathophysiology of rheumatoid arthritis.. Eur J Immunol.

[13] Ardoin SP, Pisetsky DS (2008). The role of cell death in the pathogenesis of autoimmune disease: HMGB1 and microparticles as intercellular mediators of inflammation.. Mod Rheumatol.

[14] Hugel B, Martinez MC, Kunzelmann C, Freyssinet JM (2005). Membrane microparticles: two sides of the coin.. Physiology.

[15] Chironi GN, Boulanger CM, Simon A, Dignat-George F, Freyssinet JM (2009). Endothelial microparticles in diseases.. Cell Tissue Res.

[16] Combes V, Simon AC, Grau GE, Arnoux D, Camoin L (1999). In vitro generation of endothelial microparticles and possible prothrombotic activity in patients with lupus anticoagulant.. J Clin Invest.

[17] Brogan PA, Shah V, Brachet C, Harnden A, Mant D (2004). Endothelial and platelet microparticles in vasculitis of the young.. Arthritis Rheum.

[18] Minagar A, Jy W, Jimenez JJ, Sheremata WA, Mauro LM (2001). Elevated plasma endothelial microparticles in multiple sclerosis.. Neurology.

[19] Knijff-Dutmer EA, Koerts J, Nieuwland R, Kalsbeek-Batenburg EM, van de Laar MA (2002). Elevated levels of platelet microparticles are associated with disease activity in rheumatoid arthritis.. Arthritis Rheum.

[20] Berckmans RJ, Nieuwland R, Tak PP, Boing AN, Romijn FP (2002). Cell-derived microparticles in synovial fluid from inflamed arthritic joints support coagulation exclusively via a factor VII-dependent mechanism.. Arthritis Rheum.

[21] Berckmans RJ, Nieuwland R, Kraan MC, Schaap MCL, Pots D (2005). Synovial microparticles from arthritic patients modulate chemokine and cytokine release by synoviocytes.. Arthritis Res Ther.

[22] Distler JH, Jungel A, Huber LC, Seemayer CA, Reich CF, III (2005). The induction of matrix metalloproteinase and cytokine expression in synovial fibroblasts stimulated with immune cell microparticles.. Proc Natl Acad Sci U S A.

[23] Larkin M (2001). Raised endothelial microparticles an early marker for multiple sclerosis?. Lancet.

[24] Sheremata WA, Jy W, Delgado S, Minagar A, McLarty J (2006). Interferon-β-1a reduces plasma CD31+ endothelial microparticles (CD31+EMP) in multiple sclerosis.. J Neuroinflammation.

[25] Jimenez J, Jy W, Mauro LM, Horstman LL, Ahn ER (2005). Elevated endothelial microparticle-monocyte complexes induced by multiple sclerosis plasma and the inhibitory effects of interferon-β 1b on release of endothelial microparticles, formation and transendothelial migration of monocyte-endothelial microparticle complexes.. Mult Scler.

[26] Jy W, Minagar A, Jimenez JJ, Sheremata WA, Mauro LM (2004). Endothelial microparticles (EMP) bind and activate monocytes: elevated EMP-monocyte conjugates in multiple sclerosis.. Front Biosci.

[27] Messer L, Alsaleh G, Freyssinet JM, Zobairi F, Leray I (2009). Microparticle-induced release of B-lymphocyte regulators by rheumatoid synoviocytes.. Arthritis Res Ther.

[28] Boilard E, Nigrovic PA, Larabee K, Watts GF, Coblyn JS (2010). Platelets amplify inflammation in arthritis via collagen-dependent microparticle production.. Science.

[29] Scanu A, Molnarfi N, Brandt KJ, Gruaz L, Dayer JM (2008). Stimulated T cells generate microparticles, which mimic cellular contact activation of human monocytes: differential regulation of pro- and anti-inflammatory cytokine production by high-density lipoproteins.. J Leukoc Biol.

[30] Hyka N, Dayer JM, Modoux C, Kohno T, Edwards CK3 (2001). Apolipoprotein A-I inhibits the production of interleukin-1β and tumor necrosis factor-α by blocking contact-mediated activation of monocytes by T lymphocytes.. Blood.

[31] Bresnihan B, Gogarty M, Fitzgerald O, Dayer JM, Burger D (2004). Apolipoprotein A-I infiltration in rheumatoid arthritis synovial tissue: a control mechanism of cytokine production?. Arthritis Res Ther.

[32] Gruaz L, Delucinge-Vivier C, Descombes P, Dayer JM, Burger D (2010). Blockade of T cell contact-activation of human monocytes by high-density lipoproteins reveals a new pattern of cytokine and inflammatory genes.. PLoS ONE.

[33] Jungo F, Dayer JM, Modoux C, Hyka N, Burger D (2001). IFN-β inhibits the ability of T lymphocytes to induce TNF-α and IL-1β production in monocytes upon direct cell-cell contact.. Cytokine.

[34] Sabatier F, Roux V, Anfosso F, Camoin L, Sampol J (2002). Interaction of endothelial microparticles with monocytic cells in vitro induces tissue factor-dependent procoagulant activity.. Blood.

[35] Nomura S, Tandon NN, Nakamura T, Cone J, Fukuhara S (2001). High-shear-stress-induced activation of platelets and microparticles enhances expression of cell adhesion molecules in THP-1 and endothelial cells.. Atherosclerosis.

[36] Banfi C, Brioschi M, Wait R, Begum S, Gianazza E (2005). Proteome of endothelial cell-derived procoagulant microparticles.. Proteomics.

[37] Pluskota E, Woody NM, Szpak D, Ballantyne CM, Soloviev DA (2008). Expression, activation, and function of integrin αMβ2 (Mac-1) on neutrophil-derived microparticles.. Blood.

[38] Deshmane SL, Kremlev S, Amini S, Sawaya BE (2009). Monocyte chemoattractant protein-1 (MCP-1): an overview.. J Interferon Cytokine Res.

[39] Annunziato F, Romagnani S (2009). Heterogeneity of human effector CD4+ T cells.. Arthritis Res Ther.

[40] Pisetsky DS (2009). Microparticles as biomarkers in autoimmunity: from dust bin to center stage.. Arthritis Res Ther.

[41] Burger D, Molnarfi N, Gruaz L, Dayer JM (2004). Differential induction of IL-1β and TNF by CD40 ligand or cellular contact with stimulated T cells depends on the maturation stage of human monocytes.. J Immunol.

[42] Molnarfi N, Gruaz L, Dayer JM, Burger D (2007). Opposite regulation of IL-1β and secreted IL-1 receptor antagonist production by phosphatidylinositide-3 kinases in human monocytes activated by lipopolysaccharides or contact with T cells.. J Immunol.

[43] Faille D, Combes V, Mitchell AJ, Fontaine A, Juhan-Vague I (2009). Platelet microparticles: a new player in malaria parasite cytoadherence to human brain endothelium.. FASEB J.

[44] Havel RJ, Eder HA, Bragton JH (1955). The distribution and chemical composition of ultracentrifugally separated lipoproteins in human serum.. J Clin Invest.

